# Efficacy of permissive underfeeding for critically ill patients: an updated systematic review and trial sequential meta-analysis

**DOI:** 10.1186/s40560-024-00717-3

**Published:** 2024-01-23

**Authors:** Han-yang Yue, Wei Peng, Jun Zeng, Yang Zhang, Yu Wang, Hua Jiang

**Affiliations:** 1 Institute for Emergency and Disaster Medicine, Sichuan Academy of Medical Science, Sichuan Provincial People’s Hospital, School of Medicine, University of Electronic Science and Technology of China, Chengdu, 610072 China; 2 Sichuan Provincial Research Center for Emergency Medicine and Critical Illness, Sichuan Academy of Medical Science, Sichuan Provincial People’s Hospital, School of Medicine, University of Electronic Science and Technology of China, Chengdu, 610072 China; 3 Department of Clinical Nutrition, Department of Health Medicine, Peking Union Medical College Hospital, Chinese Academy of Medical Sciences and Peking Union Medical College, No. 1 Shuai Fu Yuan Wang Fu Jing, Dong Cheng District, Beijing, 100730 China

**Keywords:** Permissive underfeeding, Hypocaloric, Low calorie, Critically ill, Meta-analysis, Systematic review, Trial sequential analyses (TSA)

## Abstract

**Background:**

Our previous study in 2011 concluded that permissive underfeeding may improve outcomes in patients receiving parenteral nutrition therapy. This conclusion was tentative, given the small sample size. We conducted the present systematic review and trial sequential meta-analysis to update the status of permissive underfeeding in patients who were admitted to the intensive care unit (ICU).

**Methods:**

Seven databases were searched: PubMed, Embase, Web of Science, China National Knowledge Infrastructure, Wanfang, Chinese Biomedical Literature Database, and Cochrane Library. Randomized controlled trials (RCTs) were included. The Revised Cochrane risk-of-bias tool (ROB 2) was used to assess the risk of bias in the enrolled trials. RevMan software was used for data synthesis. Trial sequential analyses (TSA) of overall and ICU mortalities were performed.

**Results:**

Twenty-three RCTs involving 11,444 critically ill patients were included. There were no significant differences in overall mortality, hospital mortality, length of hospital stays, and incidence of overall infection. Compared with the control group, permissive underfeeding significantly reduced ICU mortality (risk ratio [RR] = 0.90; 95% confidence interval [CI], [0.81, 0.99]; P = 0.02; I^2^ = 0%), and the incidence of gastrointestinal adverse events decreased (RR = 0.79; 95% CI, [0.69, 0.90]; P = 0.0003; I^2^ = 56%). Furthermore, mechanical ventilation duration was reduced (mean difference (MD) = − 1.85 days; 95% CI, [− 3.44, − 0.27]; P = 0.02; I^2^ = 0%).

**Conclusions:**

Permissive underfeeding may reduce ICU mortality in critically ill patients and help to shorten mechanical ventilation duration, but the overall mortality is not improved. Owing to the sample size and patient heterogeneity, the conclusions still need to be verified by well-designed, large-scale RCTs.

*Trial Registration* The protocol for our meta-analysis and systematic review was registered and recorded in PROSPERO (registration no. CRD42023451308). Registered 14 August 2023

**Supplementary Information:**

The online version contains supplementary material available at 10.1186/s40560-024-00717-3.

## Background

Nutritional therapy plays a pivotal role in critical care [1, 2]. Malnutrition in critically ill patients is related to prolonged stay in the intensive care unit (ICU), increased complications, and even associated with elevated risk of death [2, 3]. Appropriate nutritional intake helps critically ill patients maintain immune functions. It may also improve the hypercatabolic status and reduce occurrence of malnutrition, resulting in better clinical outcomes [1, 4].

According to the latest guidelines for nutrition in the ICU by the European Society for Clinical Nutrition and Metabolism (ESPEN), hypocaloric nutrition (not exceeding 70% of energy expenditure) in the early phase of acute illness limits the occurrence of overfeeding and other adverse outcomes [5]. Overfeeding is associated with complications such as hepatic steatosis and increased respiratory efforts, which have adverse effects on clinical outcomes [1, 6]. A recent study of 1,206 patients in 26 ICUs found that early high-energy feeding was detrimental in critically ill patients [7]. After decades of exploration, permissive underfeeding is a solution that balances the benefits of nutritional support with the adverse effects of overfeeding during the early stage of ICU admission [7–11]. In 2011, our team conducted a systematic review of randomized controlled trials (RCTs) to evaluate the clinical efficacy of hypocaloric nutrition in patients who received parenteral nutrition. Although we concluded that hypocaloric parenteral nutrition may shorten the length of hospital stay (LOS) and reduce the incidence of infection, the study was limited by its relatively small number of patients, with only 359 participants [12]. Subsequently, multiple well-designed studies that provided more data on permissive underfeeding in critically ill patients were published [9, 13]. Therefore, it is necessary to conduct a new systematic review and meta-analysis to update this evidence. In addition, trial-sequential meta-analysis techniques have emerged that can help researchers evaluate the power of evidence. Therefore, we conducted a systematic review and a trial sequential meta-analysis to evaluate the efficacy of permissive underfeeding in critically ill patients.

## Methods

### Protocol and registration

We designed and conducted this systematic review and meta-analysis following the PRISMA (Preferred Reporting Items for Systematic Reviews and Meta-Analysis) guidelines. The protocol for our meta-analysis and systematic review was registered and recorded in PROSPERO (registration no. CRD42023451308, registered 14 August 2023).

### Inclusion criteria

The inclusion criteria were established following the PICOS method, as outlined below:

- P (Participants): Adult patients (age ≥ 18 years) admitted to the ICU with APACHE II scores ≥ 10 points
- I (Intervention): Permissive underfeeding (actual calorie intake < 70% of target calorie or < 20 kcal/kg. d)
- C (Comparison): Administration of isocaloric feeding (actual calorie intake ≥ 70% of target calorie or ≥ 20 kcal/kg. d)
- O (Outcomes): Primary outcomes: overall mortality; secondary outcomes: duration of mechanical ventilation (days), ICU mortality, in-hospital mortality, length of hospital stay (days), incidence of overall infection, incidence of gastrointestinal adverse events
- S (Study design): Randomized controlled trials

### Exclusion criteria

The exclusion criteria encompassed the following: (a) post hoc analysis of a randomized controlled trial, (b) crossover randomized trial, (c) studies that did not address any primary or secondary outcomes, (d) pregnant or lactating women, (e) patients receiving previous nutritional support during the same hospitalization period, (f) studies involving transplantation programs, and (g) those specifically focused on cancer patients.

### Literature sources and retrieval strategy

To achieve a thorough search of studies, two reviewers (YHY and WP) independently searched seven databases: PubMed, Embase, Web of Science, China National Knowledge Infrastructure (CNKI), Wanfang, Chinese Biomedical Literature Database (SinoMed), and Cochrane Library. RCTs published before October 31, 2023, that met the inclusion criteria were included for further analysis. The retrieval process is summarized in Additional file 1: Table S1.

### Literature screening and data extraction

To avoid errors and missing data, two reviewers (YHY and WP) independently conducted literature screening and data extraction following the PRISMA guidelines. Controversies were initially managed using guidelines and discussed by the research team. If an initial resolution was not achieved, a third senior reviewer (JH) was consulted. The data extracted included study design, baseline patient information, statistics on ICU mortality, overall mortality, duration of mechanical ventilation (days), in-hospital mortality, length of hospital stay (days), incidence of overall infection, incidence of gastrointestinal adverse events.

### Assessment of risk of bias

To guarantee the reliability of this study, two reviewers (YHY and JZ) independently assessed the risk of bias in the enrolled studies. The Revised Cochrane risk-of-bias tool (ROB 2) was used to assess the risk of bias in the RCTs. RCTs cover five dimensions of bias that can affect quality: bias arising from the randomization process, deviations from intended interventions, missing outcome data, outcome measurements, and selection of the reported result. Controversies were initially managed through discussion within the research team. If an initial resolution was not achieved, a third senior reviewer (JH) was consulted.

### Statistical analyses

The Cochrane Collaboration’s Review Manager (RevMan) version 5.4 was used to pool the effects of interventions. Dichotomous variables were pooled and presented as risk ratios (RR) and 95% confidence intervals (CI) using the Mantel–Haenszel method. Continuous variables were pooled and presented as mean difference (MD) and 95% CI using the inverse variance method. The statistically significant level α was set at 0.05. Statistical differences were considered significant at P < 0.05. Statistical heterogeneity existed if I^2^ ≠ 0, and heterogeneity of pooled results was considered high if I^2^ > 50%. Random-effects models were used when heterogeneity was observed (I^2^ > 0) [14]; however, in cases where I^2^ was equal to 0, a fixed-effects model was used instead. Subgroup analysis was performed if the types of patients and interventions included in the studies were not identical. Publication bias was assessed only when the number of enrolled studies exceeded 10 because a limited number of studies undermined the power of the tests. Finally, sensitivity analysis was conducted to evaluate the reliability and authenticity of the results.

### Trial sequential analysis

An updated meta-analysis with new RCTs may lead to false-positive results because sparse data increase the risk of random error. Trial sequential analyses (TSA) reduced the risk of random errors arising from inadequate sample sizes or repetitive tests and helped in estimating the required information size (RIS) for meta-analysis. We performed TSA for outcomes using TSA version 0.9.5.10 Beta software. Type 1 error and power were set to 5% and 80%, respectively.

### Certainty and importance of evidence

The online tool GRADEpro, developed by the Grading of Recommendations Assessment, Development, and Evaluation (GRADE) Working Group, was used to evaluate the certainty and importance of the evidence [15]. The following items were individually rated: study design, risk of bias, imprecision, indirectness, and inconsistency. In accordance with the guidelines, the certainty for evidence was rated as “High, Moderate, Low, or Very low” by use of GRADEpro. And the importance of outcome was scored and categorized into one of three levels: “not important,” “important,” and “critical.”

## Results

### Literature retrieval results and characteristics

**Fig. 1:**
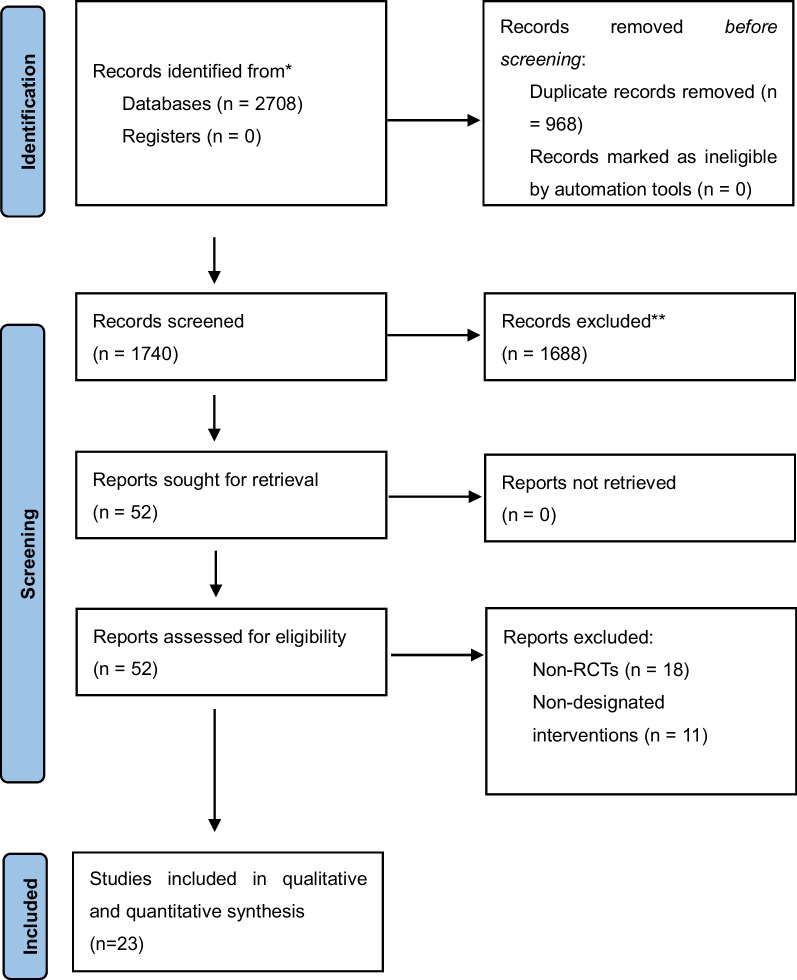
Flowchart of literature search and screening process. *Name of database and number of studies searched: PubMed (n = 654); Embase (n = 969); Web of Science (n = 58); Cochrane Library (n = 840); SinoMed (n = 167); CNKI (n = 5); WANFANG Data (n = 15)

Initially 2708 records were retrieved. After removing duplicates, screening titles and abstracts, 52 studies were retained for full-text screening. Twenty-three RCTs involving 11,444 critically ill patients were included [9, 11, 13, 16–35]. The sample sizes of the included RCTs ranged from 16 to 3957. The mean Body Mass Index (BMI) of 14 RCTs was higher than the normal range, 25 kg/m^2^. The basic characteristics of the included RCTs are presented in Additional file 2: Table S2. A flowchart of the literature search and screening process is shown in Fig. 1.

### Assessment of risk of bias

**Fig. 2:**
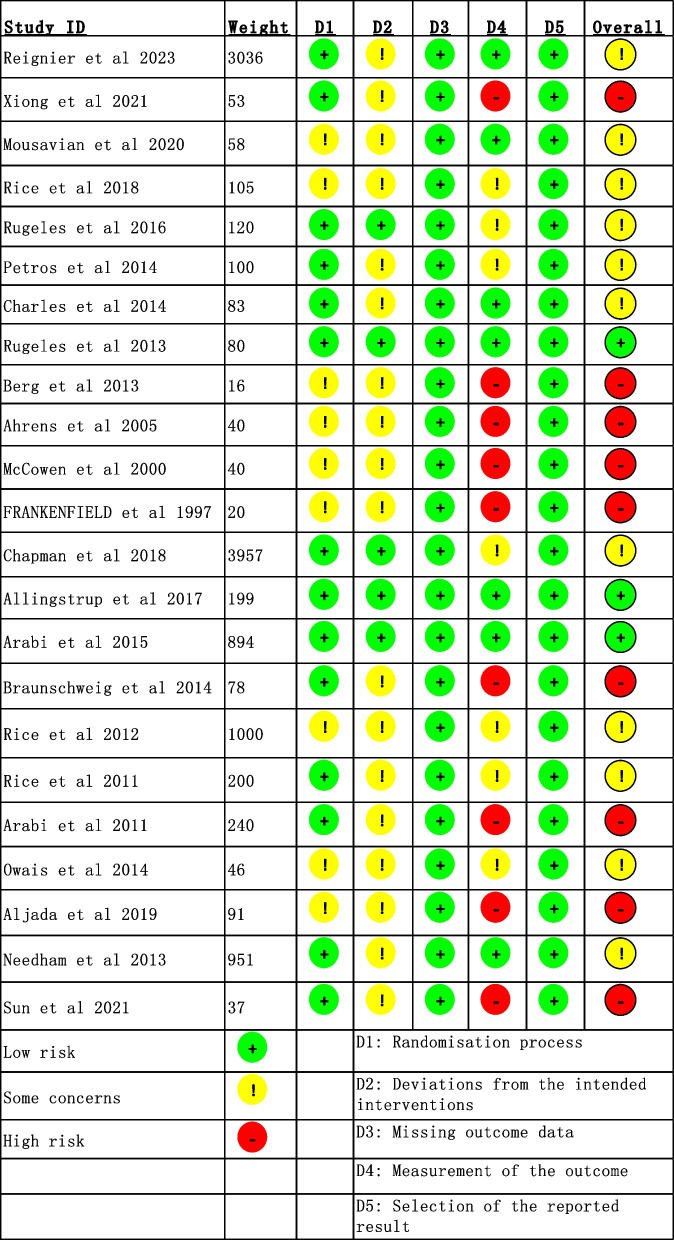
Reviewing authors’ judgments for each risk of bias item in included studies

According to the criteria of ROB 2, three RCTs were considered high quality. ROB 2 indicated high risk for nine RCTs because they did not report the measurement of the outcome. Plots of the risk of bias among the 23 RCTs are demonstrated in Fig. 2 and Additional file 3. Fig S1.

### Primary outcomes

#### Overall mortality

**Fig. 3:**
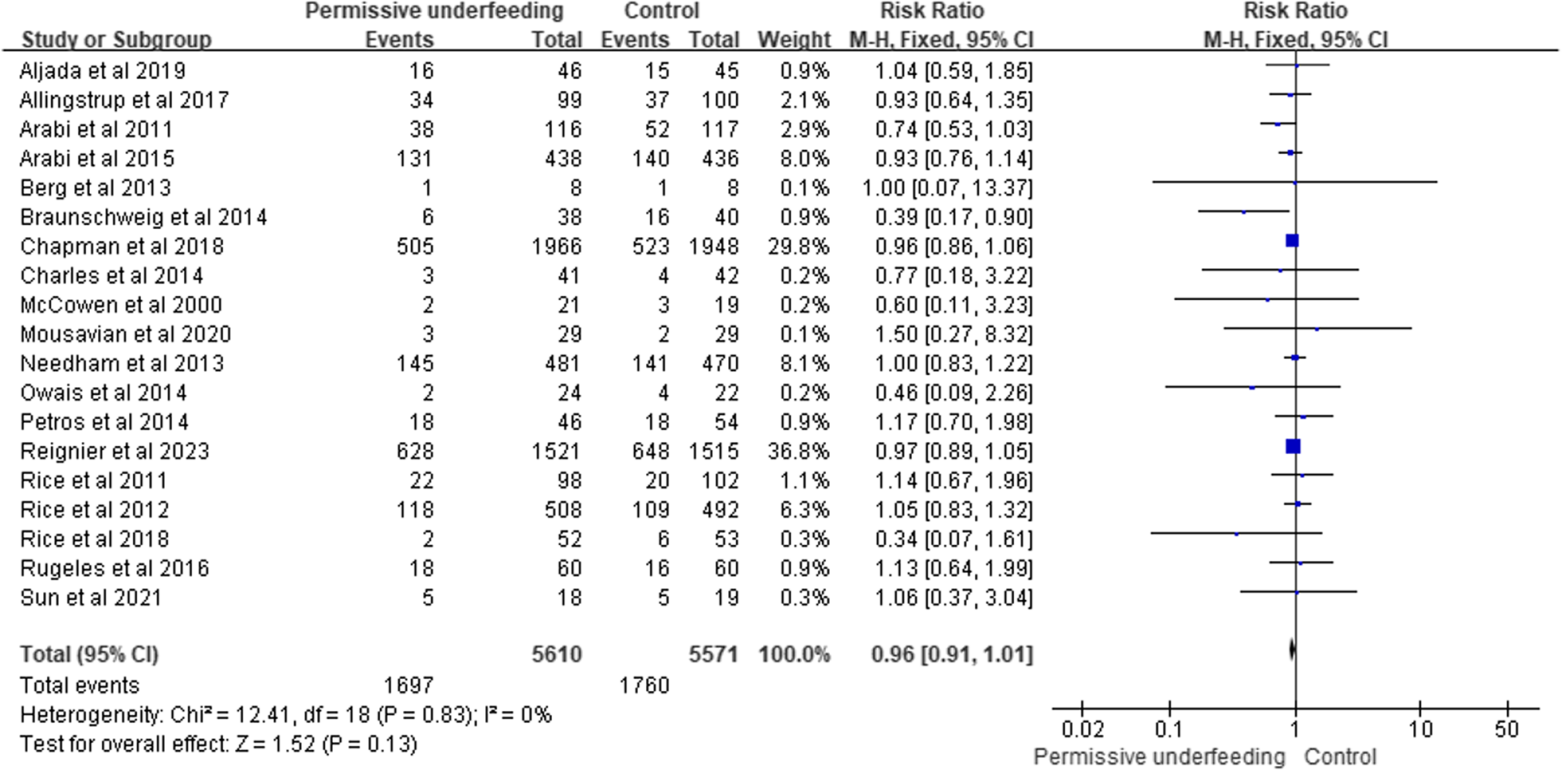
Forest plot for the comparison of overall mortality

Nineteen RCTs, including 11,181 patients, reported overall mortality [9, 11, 13, 17, 19–22, 24–33, 35]. The pooled data indicated that there was no statistically significant difference in overall mortality between the two groups (RR = 0.96; 95% CI, [0.91, 1.01]; P = 0.13) without heterogeneity (I^2^ = 0%). The certainty of the evidence was deemed moderate. A forest plot of overall mortality is shown in Fig. 3.

### Secondary outcomes

#### ICU mortality

Five RCTs including 4,361 patients reported ICU mortality [9, 19, 25, 28, 32]. Pooled data indicated that permissive underfeeding in critically ill patients was associated with lower ICU mortality than in the control group (RR = 0.90; 95% CI, [0.81, 0.99]; P = 0.02) without heterogeneity (I^2^ = 0%). The certainty of the evidence was deemed moderate. A forest plot of ICU mortality is shown in Additional file 3. Fig S2.

#### Duration of mechanical ventilation (days)

Three RCTs, including 411 patients, reported the duration of mechanical ventilation [19, 23, 32]. Pooled data indicated that permissive underfeeding in critically ill patients was significantly associated with shorter mechanical ventilation durations (MD = − 1.85 days; 95% CI, [− 3.44, − 0.27]; P = 0.02) without heterogeneity (I^2^ = 0%). The certainty of the evidence was deemed moderate. A forest plot of the mechanical ventilation duration is shown in Additional file 3: Fig S3.

#### In-hospital mortality

Nine RCTs, including 9563 patients, reported in-hospital mortality [9, 13, 19, 20, 24, 25, 28, 31, 32]. The pooled data indicated no statistically significant difference in hospital mortality between the two groups (RR = 0.95; 95% CI, [0.89, 1.02]; P = 0.18; I^2^ = 1%). The certainty of the evidence was deemed moderate. A forest plot of the in-hospital mortality rates is shown in Additional file 3: Fig S4.

#### Length of hospital stay (days)

Six RCTs, including 637 patients, reported the length of hospital stay [11, 17, 19, 26, 31, 32]. The pooled data indicated no statistically significant difference in the length of hospital stay between the two groups (MD = 1.11; 95% CI [− 2.16, 4.38]; P = 0.51; I^2^ = 77%). The certainty of the evidence was deemed low. A forest plot of length of hospital stay is shown in Additional file 3: Fig S5.

#### Incidence of overall infection

Fourteen RCTs, including 9782 patients, reported the incidence of overall infection [9, 11, 13, 17, 18, 20, 21, 25–28, 30, 33, 34]. The pooled data indicated no statistically significant difference in the incidence of overall infection between the two groups (RR = 0.92; 95% CI, [0.79, 1.06]; P = 0.25; I^2^ = 49%). The certainty of the evidence was deemed moderate. A forest plot of the incidence of overall infection is shown in Additional file 3: Fig S6.

#### Incidence of gastrointestinal adverse events

Nine RCTs, including 8423 patients, reported the incidence of gastrointestinal adverse events [9, 13, 21, 25, 28, 29, 33–35]. Pooled data indicated that permissive underfeeding in critically ill patients was significantly associated with a lower incidence of adverse gastrointestinal events in both groups (RR = 0.79; 95% CI, [0.69, 0.90]; P = 0.0003; I^2^ = 56%). The certainty of the evidence was deemed high. A forest plot of the incidence of gastrointestinal adverse events is shown in Additional file 3: Fig S7.

### Subgroup analysis

Based on the various intervention periods, we performed subgroup analyses of overall mortality, in-hospital mortality, and incidence of overall infection. After excluding studies that did not report intervention periods, pooled data showed that no significant differences in overall mortality (RR = 1.01; 95% CI, [0.88, 1.16]; P = 0.89; I^2^ = 0%), in-hospital mortality (RR = 1.06; 95% CI, [0.82, 1.37]; P = 0.66; I^2^ = 8%), and incidence of overall infection (RR = 0.83; 95% CI, [0.48, 1.43]; P = 0.50; I^2^ = 75%) between the two groups in RCTs with an intervention period of < 7 days. Likewise, pooled data showed no significant differences in overall mortality (RR = 0.96; 95% CI, [0.90, 1.02]; P = 0.16; I^2^ = 0%), in-hospital mortality (RR = 0.96; 95% CI, [0.89, 1.03]; P = 0.22; I^2^ = 0%), and incidence of overall infection (RR = 0.93; 95% CI, [0.81, 1.07]; P = 0.30; I^2^ = 41%) between the two groups in RCTs with an intervention period of ≥ 7 days. Details of the subgroup analysis are shown in Additional file 3: Fig S8-10.

### Publication bias

Funnel plots used to evaluate publication bias were symmetrical, indicating that no publication bias was observed. Funnel plots of the outcomes reported in more than 10 studies are shown in Additional file 3: Fig S11-12.

### Sensitivity analysis

Sensitivity analysis was conducted to assess the stability of outcomes. Regarding overall mortality, the exclusion of individual studies did not have an impact on the results when compared with the pooled result. Regarding ICU mortality, the exclusion of the study by Reignier et al. [9] changed the results compared with the pooled results. Regarding in-hospital mortality, the exclusion of individual studies did not have an impact on the results when compared with pooled results. The details are shown in Additional file 3: Fig S13-15.

### Trial sequential analysis

**Fig. 4:**
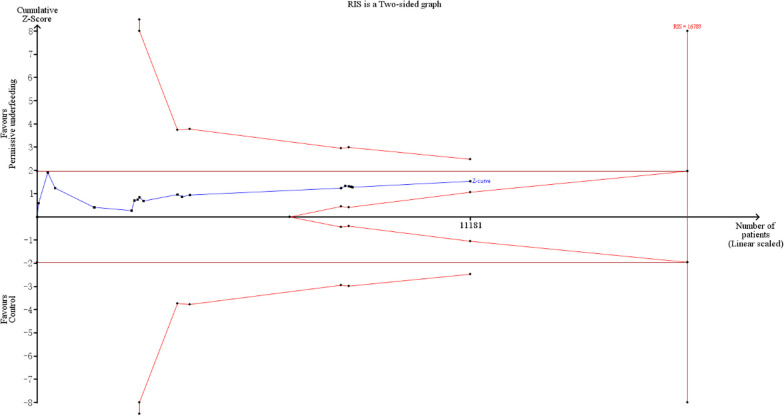
Trial sequential analysis for overall mortality

**Fig. 5:**
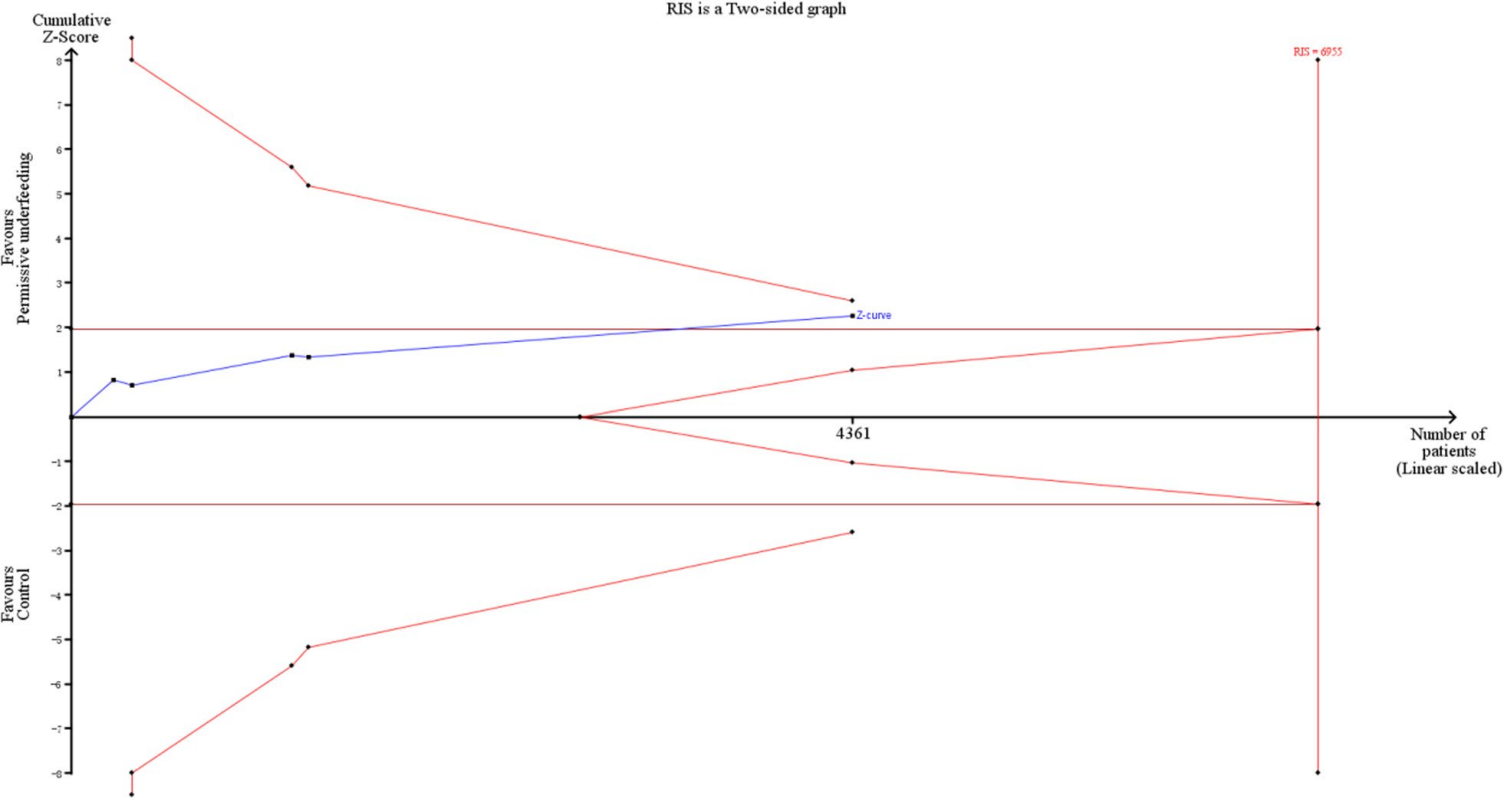
Trial sequential analysis for ICU mortality

The TSA of overall mortality showed that the cumulative Z-curve crossed the no-boundary line. The RIS was estimated to be 16,789 as determined through TSA, and the cumulative Z-curve did not reach the RIS. The details are shown in Fig. 4. The TSA of ICU mortality showed that the cumulative Z-curve crossed the traditional boundary and did not cross the other boundary lines. The RIS was estimated to be 6955 as determined through TSA, and the cumulative Z-curve failed to exceed the RIS, which meant that the association between permissive underfeeding and lower ICU mortality was a false positive and more studies are needed to verify this association. The details are shown in Fig. 5.

### GRADE summary of evidence table for key outcomes

The certainty of incidence of gastrointestinal adverse events was deemed high. The certainty of evidence for ICU mortality and in-hospital mortality were rated as moderate because studies did not report whether outcome measurements were blinded. The certainty of evidence for overall mortality was rated as moderate because different studies provided inconsistent results caused by different sample sizes. The overall certainty of the evidence of this systematic review and meta-analysis was deemed moderate. The details are summarized in Table 1.

**Table 1:** GRADE summary of evidence table for key outcomes

Certainty assessment						No of patients		Effect		Certainty	Importance
No of studies	Study design	Risk of bias	Inconsis-tency	Indirec-tness	Impre-cision	Permissive underfeeding	Control	Relative (95% CI)	Absolute (95% CI)		
Length of hospital stay (day)											
6	Randomised trials	Serious^a^	Serious^b^	Not serious	Not serious	318	319	–	MD 1.11 higher (2.16 lower to 4.38 higher)	⨁⨁◯◯ Low	IMPORTANT
Overall mortality											
19	Randomised trials	Not serious	Serious^c^	Not serious	Not serious	1697/5610 (30.2%)	1760/5571 (31.6%)	RR 0.96 (0.91–1.01)	13 fewer per 1000 (from 28 fewer to 3 more)	⨁⨁⨁◯ Moderate	CRITICAL
In-hospital mortality											
9	Randomised trials	Not serious	Serious^a^	Not serious	Not serious	1266/4792 (26.4%)	1318/4771 (27.6%)	RR 0.95 (0.89–1.02)	14 fewer per 1000 (from 30 fewer to 6 more)	⨁⨁⨁◯ Moderate	CRITICAL
ICU mortality											
5	Randomised trials	Serious^a^	Not serious	Not serious	Not serious	562/2181 (25.8%)	627/2180 (28.8%)	RR 0.90 (0.81–0.99)	29 fewer per 1000 (from 55 to 3 fewer)	⨁⨁⨁◯ Moderate	CRITICAL
Incidence of overall infection											
14	Randomised trials	Serious^a^	Not serious	Not serious	Not serious	839/4904 (17.1%)	893/4878 (18.3%)	RR 0.92 (0.79–1.06)	15 fewer per 1000 (from 38 fewer to 11 more)	⨁⨁⨁◯ Moderate	IMPORTANT
Incidence of gastrointestinal adverse events											
9	Randomised trials	Not serious	Not serious	Not serious	Not serious	1720/4213 (40.8%)	2017/4210 (47.9%)	RR 0.79 (0.69–0.90)	101 fewer per 1000 (from 149 to 48 fewer)	⨁⨁⨁⨁ High	IMPORTANT
Mechanical ventilation duration (day)											
3	Randomised trials	Serious^a^	Not serious	Not serious	Not serious	206	205	–	MD 1.85 lower (3.44 lower to 0.27 lower)	⨁⨁⨁◯ Moderate	IMPORTANT

*CI* confidence interval, *MD* mean difference, *RR* risk ratio

^a^No RCTs reported if blinded to outcome measurement

^b^MD ≥ 0 in 2 RCTs while MD < 0 in 3 RCTs

^c^RR > 1 in 2 RCTs while RR ≤ 1 in 6 RCTs

## Discussion

The benefits of nutritional treatment in critically ill patients have been repeatedly demonstrated in various studies. However, the question of how to provide appropriate and personalized nutrition still presents challenges for clinical practitioners [36, 37]. The recent French-Speaking ICU Nutritional Survey (FRANS) study and revised ESPEN guidelines lean towards supporting a low-calorie approach [5, 7].

ICU mortality was lower in the underfed patients. However, overall mortality and in-hospital mortality were not significantly different between the two groups. The interpretation for the difference is that ICU mortality is a more representative outcome in the acute phase and overall/in-hospital mortality represents the long-term outcomes. In addition to the effects of nutritional interventions, there are a number of complicated factors that influence long-term outcomes. Besides, the sample size of our study was not sufficient (approximately 70% of the RIS) and may lead to a tentative result. It may change in the future by large scale trials. Our findings are not entirely consistent with those of previous studies. A meta-analysis conducted by Zhou et al. concluded that there were no benefits in terms of reducing short-term mortality or the duration of mechanical ventilation [38]. A meta-analysis conducted by Pertzov et al. concluded that isocaloric nutrition was associated with a lower 28-day mortality, and no significant difference was observed in ICU mortality between the two groups [39]. However, these two reports included fewer studies and enrolled fewer patients (1052–6986), which may cause bias in their results.

Our systematic review and meta-analysis revealed that permissive underfeeding was associated with lower ICU mortality and mechanical ventilation duration in critically ill patients. Considering the impact of the intervention period on the outcomes, we performed subgroup analyses depending on whether the intervention period was < 7 days. The results showed that regardless of whether the permissive underfeeding intervention period was < 7 days, there were no significant differences in overall mortality, in-hospital mortality, and incidence of overall infection. In our meta-analysis, early high-energy intake was associated with poor tolerance. This is consistent with the newest ESPEN guidelines [5]. Berger et al. indicated that critically ill patients who are intolerant to early full nutrition have endogenous productions of 100–300 g of glucose per day to maintain a continuous blood glucose supply to vital organs. Production is unrepressed for at least 9 days if inflammation persists. Excessive energy intake can lead to overfeeding. Intolerance to overfeeding, which results in higher mortality, is now well-demonstrated and should therefore be avoided [40]. Because of the production of endogenous glucose, the rationality of early (first 48–72 h) hypocaloric feeding in critically ill patients is sound. However, the ESPEN guidelines recommend that full nutrition (70–100%) should be prescribed progressively within 3–7 days. This recommendation was based on a meta-analysis in 2016 that compared the impact of different enteral-parenteral routes in critically ill patients [5, 41]. When taking endogenous glucose into account, we conjectured that critically ill patients may have better clinical outcomes by continuing hypocaloric feeding for the first 48–72 h rather than being given full nutrition (70–100% within 3–7 days). In our meta-analysis, continuing hypocaloric feeding during and after the first 48–72 h indeed resulted in better clinical outcomes than those in the control group. Given the limited data reported, we were unable to perform further subgroup analyses using a 3-day cutoff, and it remains unknown whether the better clinical outcomes stemmed from hypocaloric feeding during the first three days or from continued hypocaloric feeding after three days, which should be the focus of future studies. Overall hypocaloric feeding was more effective.

### Limitations

This systematic review and meta-analysis included more RCTs and sample sizes; however, yet it is still not enough to draw conclusions from the entire population of critically ill patients because most RCTs were conducted in developed countries, with only few reports were from developing countries. In high-income countries, especially the United States, patients had higher basic BMI values and the findings are therefore not generalizable to Asian and African countries. The result may not be extrapolated to malnourished patients as few patients included had a low baseline BMI. Additionally, some RCTs did not report whether they were blinded to the outcome assessment, which could increase the potential risk of bias. Outcomes such as length of hospital stay, and length of mechanical ventilation were reported by most studies while few studies reported “free days,” which limited us to conduct analysis of these more comprehensive parameters. In the subgroup analyses, we were unable to analyze all outcomes after excluding studies that did not report intervention periods because the remaining data were limited. For the same reason, we failed to conduct subgroup analyses based on the three or ten-day intervention periods. Finally, we included any route of nutritional support that is more relevant to the real world, which may also serve as a source of potential heterogeneity.

## Conclusions

Permissive underfeeding may reduce ICU mortality in critically ill patients and help to shorten mechanical ventilation duration, but the overall mortality is not improved. Owing to the sample size and patient heterogeneity, the conclusions should be further verified by well-designed, large-scale RCTs.

### Supplementary Information

**Additional file 1. Table S1:** The retrieval process.

**Additional file 2. Table S2: **Basic characteristics of included RCT.

**Additional file 3. Fig S1: **Reviewing authors’ judgements for each risk of bias item presented as percentage across all included studies. **Fig S2**: Forest plot for the comparison of ICU mortality. **Fig S3**: Forest plot for the comparison of duration of mechanical ventilation. **Fig S4**: Forest plot for the comparison of in-hospital mortality. **Fig S5**: Forest plot for the comparison of length of hospital stay. **Fig S6**: Forest plot for the comparison of incidence of overall infection. **Fig S7**: Forest plot for the comparison of incidence of gastrointestinal adverse events. **Fig S8**: Subgroup analysis of overall mortality according to intervention period. **Fig S9**: Subgroup analysis of in-hospital mortality according to intervention period. **Fig S10**: Subgroup analysis of incidence of overall infection according to intervention period. **Fig S11**: Funnel plot of overall mortality. **Fig S12**: Funnel plot of incidence of overall infection. **Fig S13**: Sensitivity analysis of overall mortality. **Fig S14**: Sensitivity analysis of ICU mortality. **Fig S15**: Sensitivity analysis of in-hospital mortality.

## Data Availability

All data and materials related to this systematic review and meta-analysis are available from the corresponding author.

## Abbreviations

LOS: Length of hospital stay
PRISMA: Preferred reporting items for systematic reviews and meta-analysis
ICU: Intensive care unit
RCTs: Randomized controlled trials
CNKI: China national knowledge infrastructure
SinoMed: Chinese biomedical literature database
ROB2: Revised Cochrane risk-of-bias tool
RevMan: Review manager
RR: Risk ratios
CI: Confidence intervals
MD: Mean difference
ESPEN: European Society for Clinical Nutrition and Metabolism
GRADE: Grading of recommendations assessment, development, and evaluation
RIS: Required information size
FRANS: French-speaking ICU nutritional survey
BMI: Body mass index
