# Supplementary material for: Efficacy of permissive underfeeding for critically ill patients: an updated systematic review and trial sequential meta-analysis
Source: J Intensive Care. 2024 Jan 23;12:4. doi: 10.1186/s40560-024-00717-3 (PMC10804832; doi:10.1186/s40560-024-00717-3)
Supplement: Supplementary file 1 — Additional file 1. Table S1: The retrieval process. [file 40560_2024_717_MOESM1_ESM.docx]

| **Database** | **Strategy** |
| --- | --- |
| **PubMed** | #3 Search: #1 AND #2  #2 Search: (((low calorie[Title/Abstract]) OR (hypocaloric[Title/Abstract])) OR (low protein[Title/Abstract])) OR (low nitrogen[Title/Abstract])  #1 Search: (((critically[Title/Abstract]) OR (critical[Title/Abstract])) OR (ventilation[Title/Abstract])) OR (ventilated[Title/Abstract]) |
| **Embase** | #7: #3 AND #6  #6: #4 OR #5  #5: critical  #4: critically  #3: #1 OR #2  #2: hypocaloric  #1: low AND ('calorie'/exp OR calorie) |
| **Web of Science** | 3: #1 AND #2  2: (((TS=(critically)) OR TS=(critical)) OR TS=(ventilation)) OR TS=(ventilated)  1: (((TS=(low calorie )) OR TS=(hypocaloric)) AND TS=(low protein)) AND TS=(low nitrogen) |
| **Cochrane Library** | #1: (low calorie):ti,ab,kw  #2: (hypocaloric):ti,ab,kw  #3: (low protein):ti,ab,kw  #4: (low nitrogen):ti,ab,kw  #5: #1or#2or#3or#4  #6: (critically):ti,ab,kw  #7: (critical):ti,ab,kw  #8: #6or#7  #9: #5and#8 |
| **SinoMed** | 5: “weizhongzheng”[quanbuziduan] AND (“didanbai”[quanbuziduan] OR “dinengliang”[quanbuziduan])  4: “weizhongzheng”[quanbuziduan]  3: “didanbai”[quanbuziduan] OR “dinengliang”[quanbuziduan]  2: “didanbai”[quanbuziduan]  1: “dinengliang”[quanbuziduan] |
| **CNKI** | (zhuti：dinengliang (jingque)) OR (zhuti：didanbai (jingque)) AND (zhuti：weizhongzheng (jingque) ) |
| **WANFANG** | (zhuti=“dinengliang”) OR zhuti=“didanbai” AND zhuti=“weizhongzheng” |

**Additional file 1. Table S1:** **The retrieval process**
