# Supplementary material for: Efficacy of permissive underfeeding for critically ill patients: an updated systematic review and trial sequential meta-analysis
Source: J Intensive Care. 2024 Jan 23;12:4. doi: 10.1186/s40560-024-00717-3 (PMC10804832; doi:10.1186/s40560-024-00717-3)
Supplement: Supplementary file 2 — Additional file 2. Table S2: Basic characteristics of included RCT. [file 40560_2024_717_MOESM2_ESM.docx]

| Study ID | Country | Study  design | Sample size | | Setting/ Patient type | Intervention | Control | Intervention  period | Age (years) | | Sex (M/F) | | BMI | |
| --- | --- | --- | --- | --- | --- | --- | --- | --- | --- | --- | --- | --- | --- | --- |
|  |  |  | P | C |  |  |  |  | P | C | P | C | P | C |
| Reignier  et al 2023 (9) | France | RCT | 1521 | 1515 | ICU | 6 kcal/kg per day | 25 kcal/kg per day | 7 days | 66±13 | 66±13 | 1010/511 | 1026/489 | 26.7  (23.0–31.1) | 27.0  (23.0–31.5) |
| Charles  et al 2014 (11) | USA | RCT | 41 | 42 | surgical ICU | 50% of the  calculated daily  caloric requirement | 100% of the  calculated daily  caloric requirement | ＞7 days | 50.4±2.8 | 53.4±2.7 | 28/13 | 31/11 | 32.9 ± 2.0 | 28.1 ± 0.9 |
| Chapman  et al 2018 (13) | Australia/ New  Zealand | RCT | 1986 | 1971 | ICU | 1.0 kcal per mL at  a dose of 1 mL per  kg of ideal body  weight per hour | 1.5 kcal per mL at  a dose of 1 mL per  kg of ideal body  weight per hour | 28 days | 57.5±16.5 | 57.2±16.6 | 1272/714 | 1221/750 | 29.3±7.9 | 29.2±7.7 |
| FRANKENFIELD  et al 1997 (16) | USA | RCT | 10 | 10 | surgical ICU | 50% of measured  energy expenditure | 100% of measured  energy expenditure | 4 days | 45±16 | 44±24 | 8/2 | 7/3 | NR | NR |
| McCowen  et al 2000 (17) | USA | RCT | 21 | 19 | adult patients | 1 L containing  1000 kcal | toward 25 kcal /kg  dry (or adjusted  ideal) weight | NR | 57.5±14.9 | 56.6±20.4 | 12/9 | 10/9 | 27.6 ± 8.1 | 25.7 ± 6.2 |
| Ahrens  et al 2005 (18) | USA | RCT | 20 | 20 | trauma center | 20 kcal per kg  per day | 30 kcal per kg  per day | NR | 45.3±17.2 | 53.1±17.9 | 15/5 | 16/4 | NR | NR |
| Arabi  et al 2011 (19) | Saudi  Arabia | RCT | 120 | 120 | surgical ICU | 60–70% of calculated requirement | 90–100% of  calculated  requirement | NR | 50.3±21.3 | 51.9±22.1 | 86/34 | 78/42 | 28.5 ± 7.4 | 28.5 ± 8.4 |
| Rice  et al 2011 (20) | USA | RCT | 98 | 102 | ICU | 10 cc/hr | feeding rate  increased  by 25 cc/hr | 6 days | 53±19 | 54±17 | 39/59 | 47/55 | 29.2 ± 10.2 | 28.2 ± 9.4 |
| Rice  et al 2012 (21) | USA | RCT | 508 | 492 | ALI | initiated at  10 mL/h | initiated at  25 mL/h | 6 days | 52±17 | 52±16 | 267/241 | 243/249 | 29.9±7.8 | 30.4±8.2 |
| Berg  et al 2013 (22) | Sweden | RCT | 8 | 8 | neurosurgical  ICU | 50% of measured  energy expenditure | 100% of measured  energy expenditure | 2 days | NR | NR | NR | NR | NR | NR |
| Rugeles  et al 2013 (23) | Colombia | RCT | 40 | 40 | ICU | 15 kcal/kg/day | 25 kcal/kg/day | 7 days | 53.3±19.5 | 55.7±19.5 | 22/18 | 24/16 | NR | NR |
| Needham  et al 2013 (24) | USA | RCT | 481 | 470 | ALI | 25% of caloric goal | 80% of caloric goal | 6 days | 52±16 | 52±15 | 254/227 | 229/241 | 30±8 | 30±8 |
| Petros  et al 2014 (25) | Germany | RCT | 46 | 54 | ICU | 50% of daily energy expenditure | 100% of daily energy expenditure | 7 days | 67.6±11.5 | 64.3±11.5 | 32/14 | 34/20 | 28.6 ± 6.5 | 27.1 ± 6.8 |
| Braunschweig  et al 2014 (26) | USA | RCT | 38 | 40 | ICU | standard nutrition  support care | >75% of estimated  energy needs per day | ＞7 days | 58.6±16.2 | 52.5±17.1 | 21/17 | 19/21 | 30.1±8.9 | 29.8±9.3 |
| Owais  et al 2014 (27) | UK | RCT | 24 | 22 | adult patients | 60% of estimated requirements | 100% of estimated requirements | ＜7 days | 69 (53-76) | 67 (54-74) | 19/5 | 13/9 | 26 (22-29) | 23 (20-28) |
| Arabi  et al 2015 (28) | Saudi  Arabia/ Canada | RCT | 448 | 446 | critically  ill patients | 40-60% of calculated caloric requirements | 70-100% of calculated caloric requirements | 14 days | 50.2±19.5 | 50.9±19.4 | 292/156 | 282/164 | 29.0±8.2 | 29.7±8.8 |
| Rugeles  et al 2016 (29) | Colombia | RCT | 60 | 60 | ICU | 15 kcal/kg per day | 25 kcal/kg per day | 7 days | 53.8±19.0 | 51.8±20.3 | 27/33 | 33/27 | median, IQR： 25 (2.5) | 25 (2.5) |
| Allingstrup  et al 2017 (30) | Denmark | RCT | 99 | 100 | ICU | standard nutrition support care | 100% of requirements | NR | 68 (52–75) | 63 (51–72) | 59/40 | 65/35 | 22 (20–25) | 22 (20–26) |
| Rice  et al 2018 (31) | USA | RCT | 52 | 53 | critically  ill patients | low-carbohydrate  (29%)EN formula | conventional  (45%) EN formula | 5 days | 61.0±14.6 | 63.3±11.9 | 30/22 | 24/29 | 33.4 ± 4.6 | 33.0 ± 5.8 |
| Aljada  et al 2019 (32) | Saudi  Arabia | RCT | 46 | 45 | critically  ill patients | 60%–70% of the  standard caloric requirement | 90%–100% of the  standard caloric requirement | NR | 50.5±20.4 | 51.8±21.3 | 33/13 | 28/17 | 29.2 ± 7.9 | 28.4 ± 8.4 |
| Mousavian  et al 2020 (33) | Iran | RCT | 29 | 29 | adult patients | started with 30%  of the daily  energy expenditure | started at 75%  of their daily  energy expenditure | 14 days | 42±14 | 40±16 | 20/9 | 21/8 | 25 ± 4.00 | 26 ± 4.04 |
| Xiong  et al 2021 (34) | China | RCT | 27 | 26 | ICU | 20–40% of ESPEN recommendations | 70–100% of ESPEN recommendations | 7 days | 49.44±14.94 | 48.31±10.41 | 20/7 | 17/9 | 21.58±1.33 | 21.95±1.40 |
| Sun  et al 2021 (35) | China | RCT | 18 | 19 | sepsis | 60% of goal  caloric requirements | 100% of goal  caloric requirements | 7 days | NR | NR | NR | NR | NR | NR |

Additional file 2. Table S2: Basic characteristics of included RCT
