# Supplementary material for: Efficacy of permissive underfeeding for critically ill patients: an updated systematic review and trial sequential meta-analysis
Source: J Intensive Care. 2024 Jan 23;12:4. doi: 10.1186/s40560-024-00717-3 (PMC10804832; doi:10.1186/s40560-024-00717-3)
Supplement: Supplementary file 3 — Additional file 3. Fig S1: Reviewing authors’ judgements for each risk of bias item presented as percentage across all included studies. Fig S2: Forest plot for the comparison of ICU mortality. Fig S3: Forest plot for the comparison of duration of mechanical ventilation. Fig S4: Forest plot for the comparison of in-hospital mortality. Fig S5: Forest plot for the comparison of length of hospital stay. Fig S6: Forest plot for the comparison of incidence of overall infection. Fig S7: Forest plot for the comparison of incidence of gastrointestinal adverse events. Fig S8: Subgroup analysis of overall mortality according to intervention period. Fig S9: Subgroup analysis of in-hospital mortality according to intervention period. Fig S10: Subgroup analysis of incidence of overall infection according to intervention period. Fig S11: Funnel plot of overall mortality. Fig S12: Funnel plot of incidence of overall infection. Fig S13: Sensitivity analysis of overall mortality. Fig S14: Sensitivity analysis of ICU mortality. Fig S15: Sensitivity analysis of in-hospital mortality. [file 40560_2024_717_MOESM3_ESM.docx]

Additional file 3. Fig S1: Reviewing authors’ judgements for each risk of bias item presented as percentage across all included studies


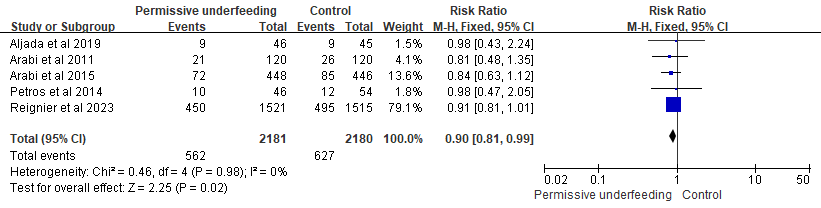


Additional file 3. Fig S2: Forest plot for the comparison of ICU mortality


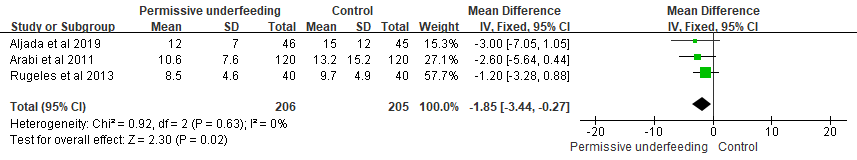


Additional file 3. Fig S3: Forest plot for the comparison of duration of mechanical ventilation


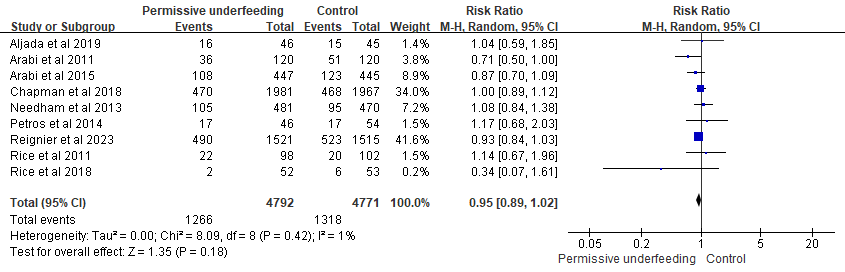


Additional file 3. Fig S4: Forest plot for the comparison of in-hospital mortality


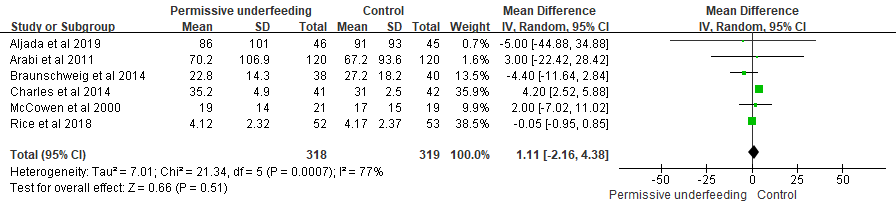


Additional file 3. Fig S5: Forest plot for the comparison of length of hospital stay


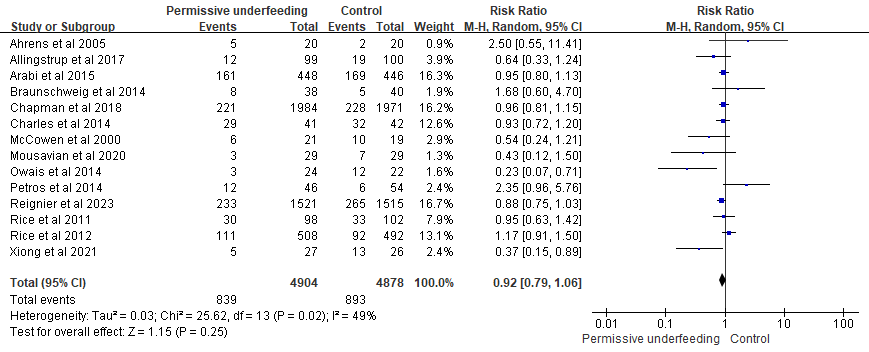


Additional file 3. Fig S6: Forest plot for the comparison of incidence of overall infection


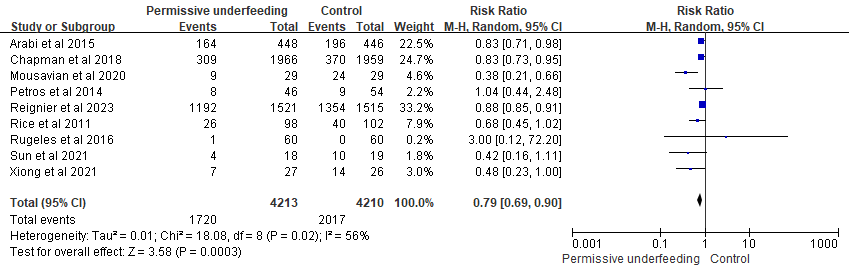


Additional file 3. Fig S7: Forest plot for the comparison of incidence of gastrointestinal adverse events


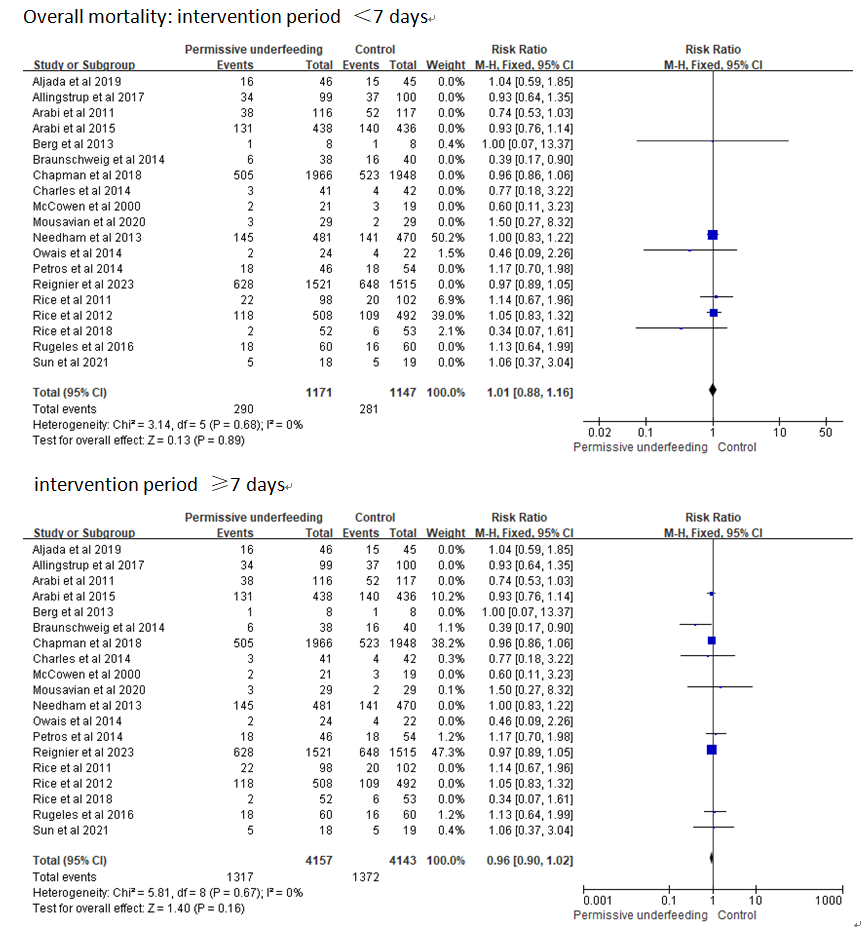


Additional file 3. Fig S8: Subgroup analysis of overall mortality according to intervention period


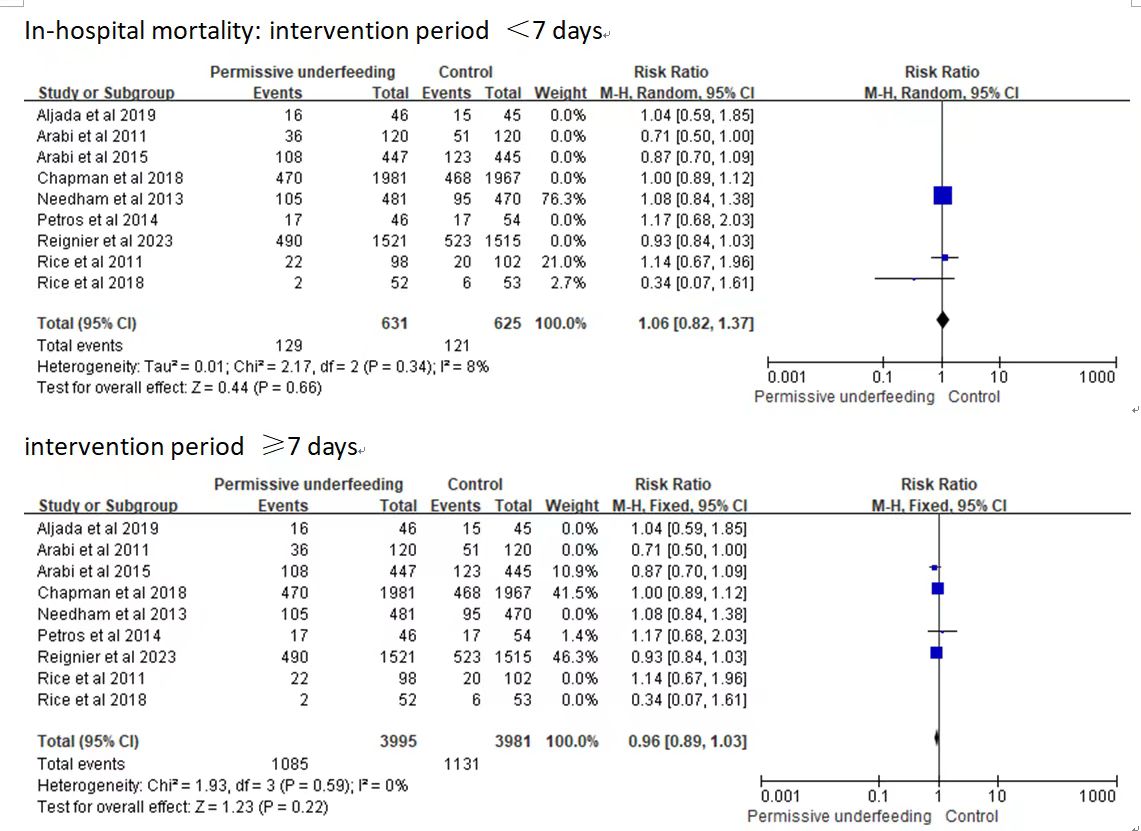


Additional file 3. Fig S9: Subgroup analysis of in-hospital mortality according to intervention period


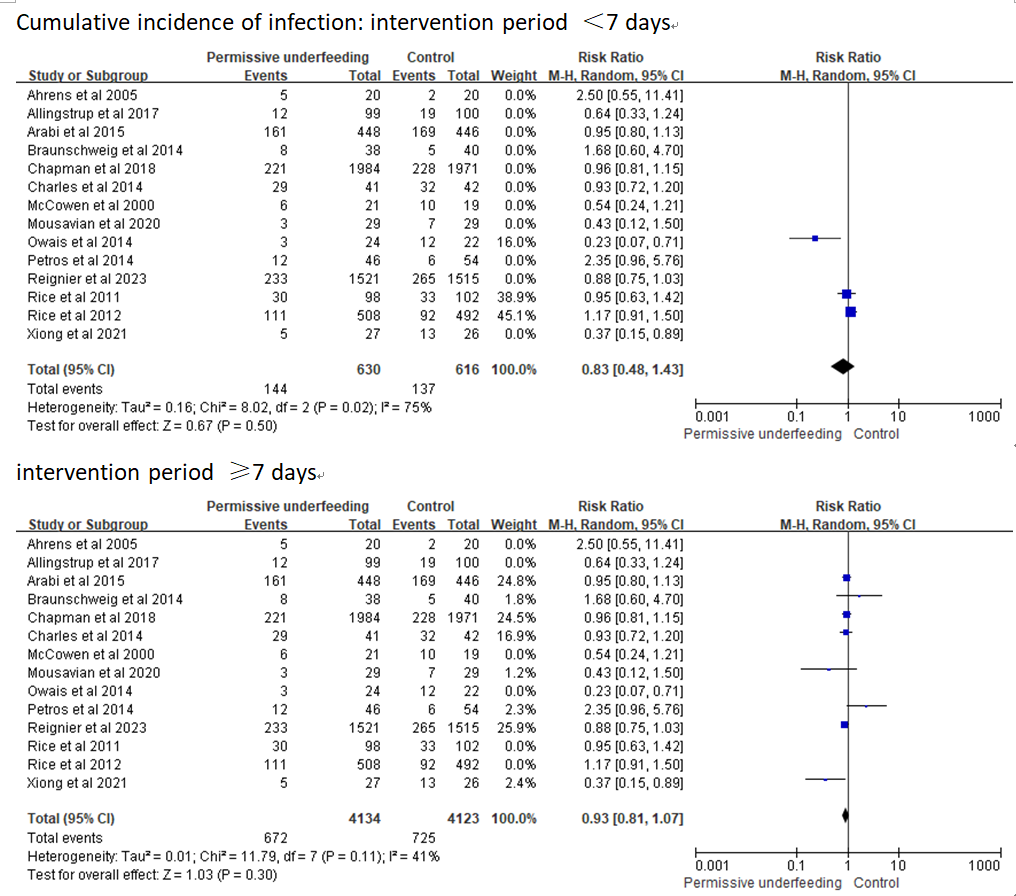


Additional file 3. Fig S10: Subgroup analysis of incidence of overall infection according to intervention period


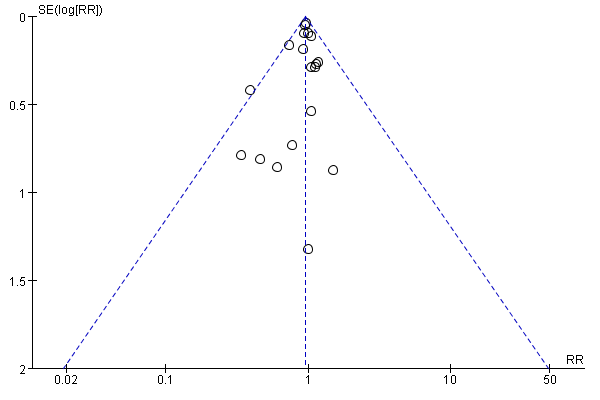


Additional file 3. Fig S11: Funnel plot of overall mortality


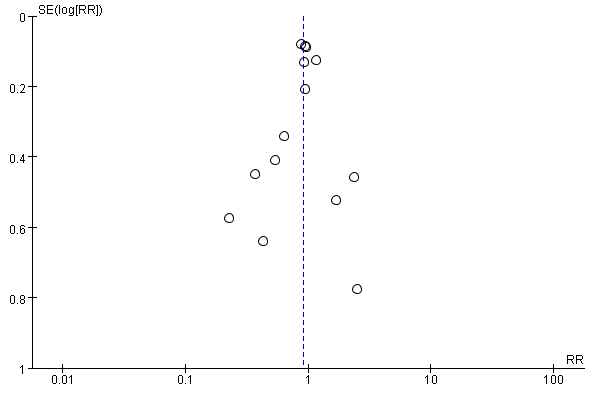


Additional file 3. Fig S12: Funnel plot of incidence of overall infection


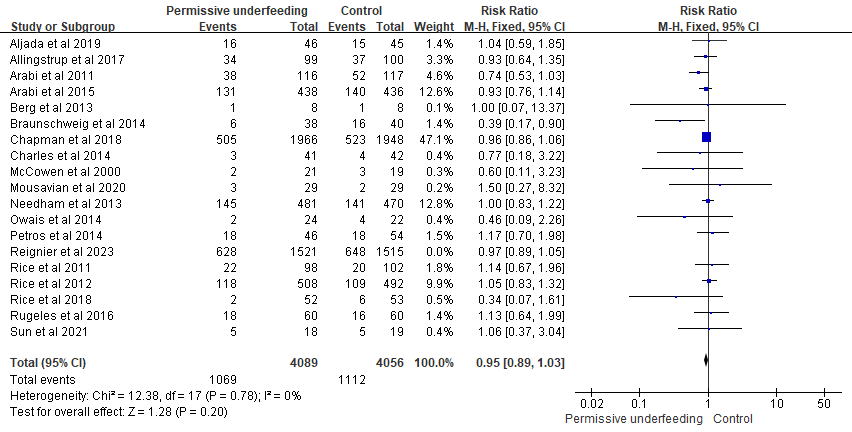


Additional file 3. Fig S13: Sensitivity analysis of overall mortality


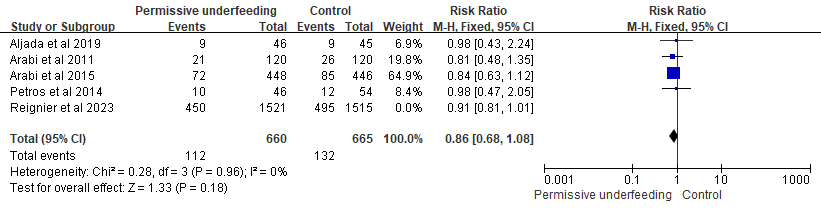


Additional file 3. Fig S14: Sensitivity analysis of ICU mortality


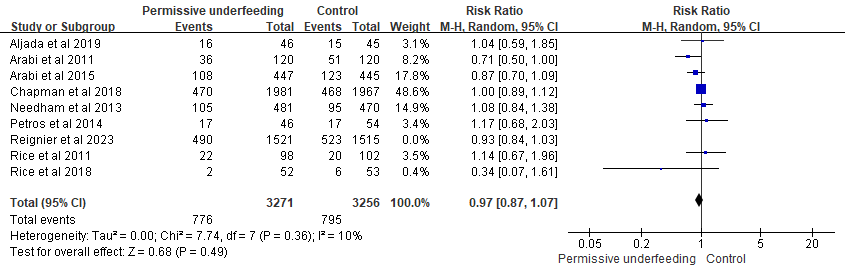


Additional file 3. Fig S15: Sensitivity analysis of in-hospital mortality
